# Immunotherapy with Monoclonal Antibodies in Lung Cancer of Mice: Oxidative Stress and Other Biological Events

**DOI:** 10.3390/cancers11091301

**Published:** 2019-09-04

**Authors:** Jun Tang, Daniel Ramis-Cabrer, Xuejie Wang, Esther Barreiro

**Affiliations:** 1 Pulmonology Department-Muscle Wasting & Cachexia in Chronic Respiratory Diseases & Lung Cancer Research Group, IMIM-*Hospital del Mar, Parc de Salut Mar*, Biomedical Research Park (PRBB), C/Dr. Aiguader, 88, E-08003 Barcelona, Spain; 2 Centro de Investigación en Red de Enfermedades Respiratorias (CIBERES), Instituto de Salud Carlos III (ISCIII), Biomedical Research Park (PRBB), C/Dr. Aiguader, 88, E-08003 Barcelona, Spain; 3 Department of Medicine, Autonomous University of Barcelona (UAB), Biomedical Research Park (PRBB), C/Dr. Aiguader, 88, E-08003 Barcelona, Spain; 4 Health and Experimental Sciences Department (CEXS), *Pompeu Fabra* University (UPF), Biomedical Research Park (PRBB), C/Dr. Aiguader, 88, E-08003 Barcelona, Spain

**Keywords:** experimental lung cancer, immunomodulators, oxidative stress, autophagy, tumor growth, sirtuin-1

## Abstract

*Background:* Lung cancer (LC) is a major leading cause of death worldwide. Immunomodulators that target several immune mechanisms have proven to reduce tumor burden in experimental models through induction of the immune microenvironment. We hypothesized that other biological mechanisms may also favor tumor burden reduction in lung cancer-bearing mice treated with immunomodulators. *Methods:* Tumor weight, area, T cells and tumor growth (immunohistochemistry), oxidative stress, apoptosis, autophagy, and signaling (NF-κB and sirtuin-1) markers were analyzed (immunoblotting) in subcutaneous tumor of BALB/c mice injected with LP07 adenocarcinoma cells treated with monoclonal antibodies (CD-137, CTLA-4, PD-1, and CD-19, *N* = 9/group) and non-treated control animals. *Results:* Compared to non-treated cancer mice, in tumors of monoclonal-treated animals, tumor area and weight and ki-67 were significantly reduced, while T cell counts, oxidative stress, apoptosis, autophagy, activated p65, and sirtuin-1 markers were increased. *Conclusions:* Immunomodulators elicited a reduction in tumor burden (reduced tumor size and weight) through decreased tumor proliferation and increased oxidative stress, apoptosis, autophagy, and signaling markers, which may have interfered with the immune profile of the tumor microenvironment. Future research should be devoted to the elucidation of the specific contribution of each biological mechanism to the reduced tumor burden.

## 1. Introduction

Lung cancer is the most prevalent cancer worldwide that affects both sexes and has a very high mortality [1]. Despite the development of new therapeutic strategies, patients with lung cancer have an overall survival rate lower than 15% in five years [1,2,3]. Respiratory conditions such as chronic obstructive pulmonary disease (COPD) and lung fibrosis predispose patients to a greater risk to develop lung cancer, especially non-small cell lung cancer (NSCLC) type [1,2,4,5,6].

The underlying biology of lung cancer is complex, as several mechanisms may interplay at different stages. For instance, inflammation, which is key in host protection, may promote lung cancer initiation and malignancy in chronic inflammatory processes such as in patients with COPD [2,5,7,8,9,10,11]. Moreover, oxidative stress was also shown to participate in tumor initiation, promotion, and progression of carcinogenesis in patients with lung cancer, particularly in those with COPD [2,9,12,13]. Besides, inflammatory events and oxidative stress may drive the release of a cascade of cytokines and growth factors, which may favor lung tumorigenesis [2,9,12,13] through interference with biological processes such as apoptosis and autophagy [11,13,14,15]. In the last few years, the implications of biological mechanisms such as increased oxidative stress, inflammatory events, particularly a Th1-predominant response, and epigenetic events were demonstrated to be differentially expressed in the lung tumors of patients with COPD compared to patients without this respiratory condition [8,11,13,16]. These results are important, since they may help establish a differential profile of patients that may be more or less susceptible to certain therapies.

The immune system defends the host against diseases, including neoplastic transformation. However, cancer cells may evade the host immune system through a process defined as cancer immunoediting [17]. Cancer immune scape results from the action of immunosuppressive pathways that involve membrane receptors that are located in immune cell types along different steps of the cancer-immunity cycle [17,18,19,20,21]. Immune checkpoints enable immune tolerance to prevent autoimmunity events in the host [17,18,19,20,21,22]. Several immune checkpoints have been identified so far. As such, programmed cell death protein 1 (PD1) is a membrane receptor that promotes immune tolerance through T cell inactivation [18,19,22]. Cytotoxic T-lymphocyte-associated protein 4 (CTLA-4) is a disruptor of antigen presentation upon T cell activation [17]. Cluster of differentiation 137 (CD137) is located in several immune cells such as T regulatory cells (Treg) that are responsible for repressing T cell activity [21]. Additionally, B cells present cluster of differentiation 19 (CD19), which can trigger pro- and anti-tumorigenic responses (Table 1) [20].

Definition of abbreviations: PD-1, programmed cell death-1; CTLA-4, cytotoxic T-lymphocyte associated protein-4; CD-137, TNF receptor superfamily member 9; CD-19; B-lymphocyte antigen; NK, natural killer.Specific immune checkpoint inhibitors have been designed, namely monoclonal antibodies that specifically act against these membrane receptors in order to boost the immune microenvironment. The blockade of these inhibitory pathways has been shown to restore the anti-tumor activity of the immune system [17,22,27,28,29,30,31,32]. The therapeutic efficacy of the combination of different immunomodulatory monoclonal antibodies has been recently demonstrated in animal models of lung cancer, in which the tumor immune microenvironment was specifically explored [18,19]. Furthermore, in previous studies from our group [8,11,12,13,16,33], the contribution of inflammation and signaling pathways [e.g., nuclear factor (NF)-kB and Sirtuin-1], oxidative stress, autophagy, and apoptosis in response to several pharmacological strategies was shown in mice bearing lung tumors. Oxidative stress was also shown to mediate the response to immunotherapy in colorectal cancer in mice [34] and the chemoresistance in ovarian cancer of patients [35]. Whether similar biological mechanisms can be observed in the tumors of mice treated with a combination of several immunomodulators remains to be identified. Thus, we reasoned that immunomodulators may also exert beneficial effects on tumor burden through biological events other than the immune microenvironment.

On this basis, we hypothesized that treatment of a combination of specific immunomodulatory monoclonal antibodies that included anti-PD1, anti-CTLA-4, anti-CD137, and anti-CD19 may have an effect on tumor progression through several biological mechanisms such as oxidative stress, autophagy, and apoptosis through specific signaling pathways in wild-type lung adenocarcinoma cells of mice [33]. Accordingly, in the current investigation, the main objectives were two-fold: (1) to assess the immune tumor microenvironment (T cells) and (2) to quantify levels of oxidative stress, antioxidant enzymes, apoptosis, autophagy, signaling, and cell proliferation rates in the subcutaneous lung adenocarcinoma tumors of BALB/c mice treated with a combination of immunomodulators (anti-PD1, anti-CTLA-4, anti-CD137, and anti-CD19 monoclonal antibodies). A group of tumor-bearing mice that did not receive treatment with the cocktail of monoclonal antibodies was the control group in the study. This experimental model of NSCLC has been previously well-validated in our group [12,33,36,37,38].

## 2. Methods

### 2.1. Animal Experiments

#### 2.1.1. Experimental Design 

**Figure 1:**
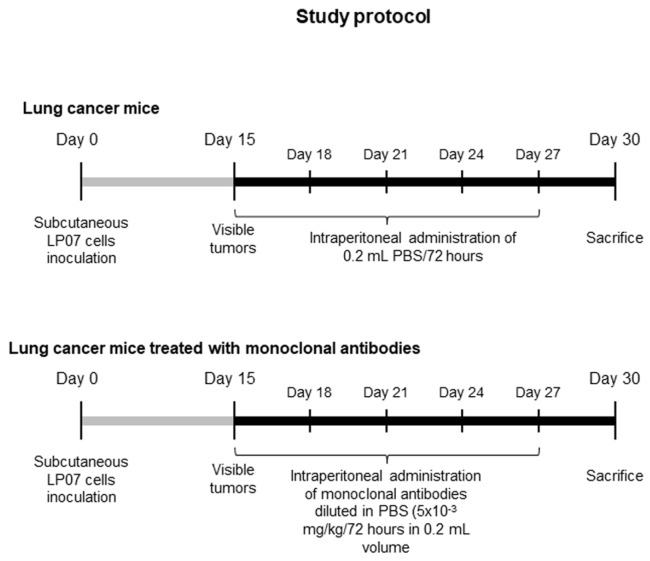
Graphical time-line representation of the non-treated control lung cancer mice and the lung cancer group of animals treated with the combination of monoclonal antibodies. This was a controlled study designed according to the ethical regulations on animal experimentation of the Spanish Legislation (Real Decreto 53/2013, BOE 34/11370–11421), the European Community Directive 2010/63/EU, and the European Convention for the Protection of Vertebrate Animals Used for Experimental and Other Scientific Purposes (1986) at PRBB. The Animal Research Committee approved all animal experiments (Animal Welfare Department in Catalonia, Spain, # EBP-15-1704).

The study protocol is illustrated in Figure 1. An animal model with lung cancer was developed through the inoculation of cancer cells from LP07 stable adenocarcinoma cell line derived from P07 lung tumor that spontaneously appeared in BALB/c mice [39,40,41]. Eighteen female BALB/c mice (8 weeks old, 20 g weight) acquired from Harlan Interfauna Ibérica SL (Barcelona, Spain) received a subcutaneous inoculation of LP07 cells (4 × 10^5^) resuspended in 0.2 mL of minimal essential medium (MEM) in the left flank (Figure 1). After tumor cell inoculation on day 0 of all the mice, they were randomly divided into two independent groups (*N* = 9/group) to be thereafter followed for 30 days: (1) experimental control group in which mice received an intraperitoneal administration of 0.2 mL phosphate-buffered saline (PBS) every 72 h (non-treated controls group) and (2) mice treated with a combination of monoclonal antibodies (treated lung cancer group) that included anti-PD1 (RMP1-14; Cat. #BE0146, BioXCell, West Lebanon, NH, USA), anti-CTLA-4 (9D9; Cat. #BE0164, BioXCell), anti-CD137 (LOB12.3; Cat. #BE0169, BioXCell), and anti-CD19 (1D3; Cat. #BE0150, BioXCell) antibodies [18,19,21,23,24,25,26] (Table 1). A dose of 5 × 10^−3^ mg/kg/72 h in 0.2 mL PBS was administered to the treated group of lung cancer mice from day 15 (tumors visible) up until day 30 (Figure 1). The intraperitoneal route was chosen in order to mimic administration of this type of therapies in clinical settings [19]. For ethical reasons we were not allowed to extend the study protocol longer than 30 days. Also for ethical reasons, only non-treated tumor-bearing mice administered with the vehicle PBS were used as the control group in the study. Food and water were supplied ad libitum and mice were kept under pathogen-free conditions with a 12:12 h light:dark cycle in the animal facilities placed in the Barcelona Biomedical Research Park (PRBB) premises.

#### 2.1.2. In Vivo Measurements Conducted on the Animals

Food intake and body weight were measured daily in all the study animals. Tumor area was also measured daily using a specific caliper in all the animals.

#### 2.1.3. Sacrifice and Sample Collection

The two experimental groups of mice were sacrificed after 30 days of inoculation of LP07 cells. In each mouse, an intraperitoneal injection of 0.1 mL sodium pentobarbital (60 mg/kg) was inoculated prior to sacrifice. In order to verify total anesthesia depth, the pedal and blink reflexes were assessed in all animals. As the histological features of the subcutaneous tumor and those of the lung metastases are identical in this LP07 mouse model of lung cancer, for practical reasons, the subcutaneous tumor was used for the laboratory experiments. As such, the subcutaneous tumor was extracted from all the mice. A fragment of the tumor specimens was immediately frozen in liquid nitrogen and stored at −80 °C, while the other fragment was immersed in an alcohol-formol to be thereafter embedded in paraffin until further use.

### 2.2. Molecular Biology Analyses

#### 2.2.1. Histological Analyses of Tumor Samples 

Immunohistochemical techniques were applied on tumor sections in order to explore expression of the proliferation marker Ki-67 and T cells, following previous methodologies [8,12,13,16,33,36,37,38,42,43]. Briefly, for all the target antigens, tumor cross-sections were deparaffinized and then antigen retrieval was carried out by heating slides in a water bath in Tris/Ethylenediaminetetraacetic acid (EDTA) buffer, pH 9, for 30 min (Ki-67 and CD3) or in a pressure-cooker (CD4, CD8) in 0.1 M citrate buffer, pH 6, for 15 min. Subsequently, samples were blocked with 3% H_2_O_2_ for 15 min, and immediately afterwards they were blocked with 1% BSA and goat serum for two hours. Slides were then incubated at room temperature for 30 min (CD3) or at 4 °C overnight (Ki-67, CD4, CD8) with the following specific primary antibodies: anti-Ki67 (Merck-Millipore, Darmstadt, Germany), anti-CD3 (DAKO, Glostrup, Denmark), anti-CD4 (Abcam, Cambridge, UK), and anti-CD8 (Abcam). Slides were washed and incubated for 30 min with biotinylated universal secondary antibody, followed by another 30 min incubation with horseradish-conjugated streptavidin and diaminobenzidine for five minutes (kit LSAB+HRP Dako Cytomation Inc., Carpinteria, CA, USA) as a substrate.

Tumor sections were counterstained with hematoxylin for two minutes and were then dehydrated and mounted for conventional microscopy. Images of the stained tumors were taken under a light microscope (Olympus BX 61, Olympus Corporation, Tokyo, Japan) coupled with a camera (Olympus DP 71, Olympus Corporation). The number of Ki-67, CD3, and CD8 cells was expressed as the percentage of the cells that were positively stained for the corresponding markers of the total cells in the tumor preparations. The number of CD4 cells was represented as the number of cells that were positively stained for CD4 in the measured area (expressed in micrometers).

*Terminal deoxynucleotidyl transferase- mediated uridine 5′-triphosphate (UTP) nick- end labelling (TUNEL) assay.* In tumor paraffin-embedded sections, apoptotic nuclei were identified using the TUNEL assay (In Situ Cell Death Detection Kit, POD, Roche Applied Science, Mannheim, Germany) for all study groups following the manufacturer’s instructions and previous studies [38,43]. Briefly, this assay is based on the principle that during the apoptosis of nuclei, genomic DNA may yield double-stranded, low molecular weight fragments as well as single-strand breaks (nicks) in high molecular weight DNA. These DNA strand breaks can be identified by labelling 3′-hydroxyl (3′OH) groups with modified nucleotides in an enzymatic reaction. In this assay, deoxynucleotidyl transferase (TdT), which catalyzes the polymerization of nucleotides to free 3′-OH DNA ends, is used to label DNA strand breaks. Briefly, diaphragm and gastrocnemius muscle sections were fixed and permeabilized. Subsequently, they were incubated with the TUNEL reaction mixture that contains terminal TdT and fluorescein-dUTP. During the incubation period, terminal TdT catalyzed the addition of fluorescein-dUTP at free 3′-OH groups in single- and double-stranded DNA. After washing, the label incorporated at the damaged sites of the DNA was marked by anti-fluorescein antibody conjugated with the reporter enzyme peroxidase. After several washes that removed unbound enzyme conjugate, the peroxidase retained in the immune complex was visualized by a substrate reaction. Apoptotic nuclei were brown, while negative nuclei were blue (hematoxylin counterstaining). In each tumor cross-section, the TUNEL-positive nuclei and the total number of nuclei were counted blindly by two independent observers, who were previously trained for that purpose. Results were expressed as the ratio of total TUNEL positively-stained nuclei to the total number of counted nuclei, as also previously reported [38,43]. A minimum amount of 300 nuclei were counted in each tumor preparation. Final results corresponded to the mean value of the counts provided by the two independent observers (concordance rate 95%). Negative control experiments, in which the TUNEL reaction mixture was omitted, were also conducted. Moreover, rat testicles were used as a positive control in these experiments.

#### 2.2.2. Immunoblotting

Protein levels of the target markers were determined using 1D electrophoresis and immunoblotting according to previously published methodologies [8,12,13,16,33,36,37,38,42]. Frozen tumor samples extracted from mice were homogenized in lysis buffer. The following specific primary antibodies were used to identify the different target markers: protein tyrosine nitration (anti-3-nitrotyrosine antibody, Invitrogen, Eugene, OR, USA), malondialdehyde protein adducts (anti-MDA protein adduct antibody, Academic Bio-Medical Company, Inc., Houston, TX, USA), catalase (anti-catalase antibody, Calbiochem, Darmstadt, Germany), Mn-superoxide dismutase (SOD2, anti-SOD2 antibody, Santa Cruz Biotechnology, Santa Cruz, CA, USA), CuZn-superoxide dismutase (SOD1, anti-SOD1 antibody, Santa Cruz), b-cell lymphoma 2 (BCL-2, anti-BCL-2, antibody Santa Cruz), BCL-2 associated X protein (BAX, anti-BAX antibody, Santa Cruz), nucleoporin p62 (anti-p62 antibody, Sigma-Aldrich, St. Louis, MO, USA), beclin-1 (anti-beclin-1 antibody, Santa Cruz), microtubule-associated protein 1 light-chain 3 (LC3B, anti-LC3B antibody, Cell Signaling, MA, USA), nuclear factor kappa-light-chain-enhancer of activated B cells (NF-κB) p65 subunit and phosphorylated p65 subunit (anti NF-κB p65 and p-NF-κB p65 antibodies, Santa Cruz), sirtuin-1 (anti-sirtuin-1 antibody, EMD Millipore, Billerica, MA, USA), and glyceraldehyde-3-phosphate dehydrogenase (GAPDH, anti-GAPDH antibody, Santa Cruz) as the protein loading control to confirm identical protein loading among different lanes. Horseradish peroxidase (HRP)-conjugated secondary antibodies and a chemiluminescence kit (Thermo Scientific, Rockford, IL, USA) were used to detect the antigens from all samples. For the sake of comparisons between the two groups, all study samples (×18) were run together in the same mini-cell electrophoresis and transfer boxes, respectively, and the corresponding membranes were detected using chemiluminescence in the same platform under identical exposure times. 

Polyvinylidene difluoride (PVDF) membranes were scanned with the Molecular Imager Chemidoc XRS System (Bio-Rad Laboratories, Hercules, CA, USA) using the software Quantity One version 4.6.5 (Bio-Rad Laboratories), and optical densities of target proteins were quantified using the software Image Lab version 2.0.1 (Bio-Rad Laboratories). Final optical densities (arbitrary units) acquired in each group of mice corresponded to the average value of all the samples (lanes). Values of optical densities (arbitrary units) of LC3B-II/LC3B-I were also calculated as the ratio of LC3B-II (14 kDa) to LC3B-I (16 kDa) protein content. Moreover, all values of the different antigens were calculated as the ratio of the optical densities of the given variable to those of the loading control GAPDH in each study sample, as shown in each figure.

Standard stripping methodologies were employed to detect p62 protein levels in the same PVDF membranes of beclin-1. The loading control GAPDH was also detected using stripping methodologies in the study markers: protein tyrosine nitration, MDA-protein adducts, SOD1, SOD2, catalase, BAX, BCL-2, beclin-1, p62, LC3B, NF-κB p65, pNF-κB p65, and Sirtuin-1. Briefly, primary and secondary antibodies were stripped off proteins using a stripping solution (25 nM glycine, pH 2.0, and 1% Sodium Dodecyl Sulphate (SDS)) for 30 min. Membranes were subsequently washed two consecutive times (10 min each) with phosphate buffered saline and tween (PBST) at room temperature. Immediately afterwards, membranes were blocked with 1% Bovine Serum Albumin (BSA) and incubated with specific primary and secondary antibodies following the abovementioned procedures.

### 2.3. Statistical Analysis

Using specific software (StudySize 2.0, CreoStat HB, Frolunda, Sweden) and assuming an alpha error of 0.05 and a minimum of 80% of standard power statistics, the sample size (*N* = 9/group) was sufficiently great to identify a difference of 700 and 0.6 points in both tumor area and weight variables between groups, respectively. The Shapiro–Wilk test was used to check the normality of the study variables. Therefore, data are expressed as mean and standard deviation in both tables and figures. The Statistical Package for the Social Sciences (SPSS, version 22, SPSS Inc., Chicago, IL, USA) was used to compare the study variables between the two study groups using the unpaired Student’s *t*-test, and statistical significance was established at *p* ≤ 0.05.

## 3. Results

### 3.1. Monoclonal Antibodies Improved Tumor Burden and Body Weight in Mice

**Figure 2:**
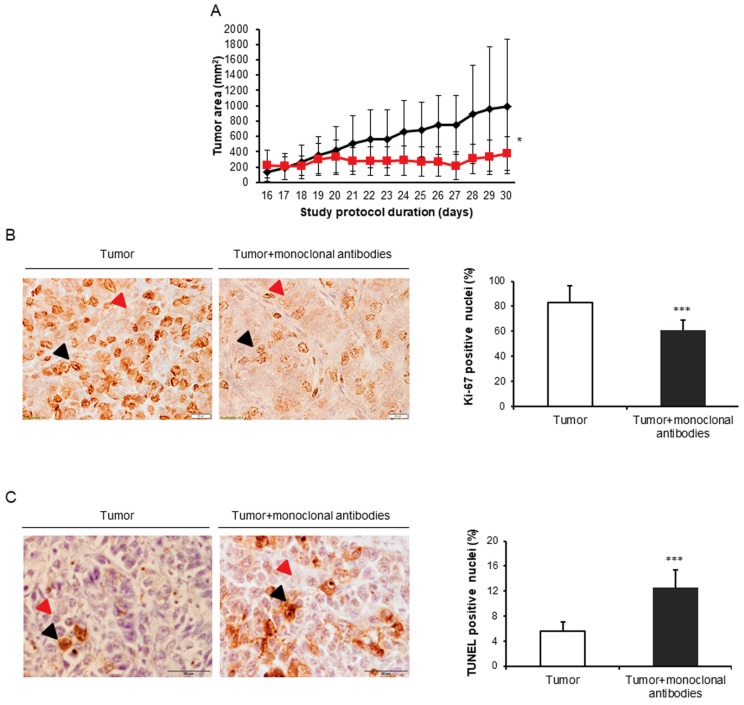
(**A**) Mean values and standard deviation of subcutaneous tumor area (mm^2^) of non-treated control lung cancer group of mice (black color) and lung cancer mice treated with monoclonal antibodies group (red color) during the study protocol. (**B**) Representative histological sections (40×) and mean values and standard deviation of Ki-67 in the subcutaneous tumors of the non-treated control mice and the treated lung cancer animals. Black arrowheads point towards Ki-67-positively stained nuclei (brown color), while red arrowheads point towards Ki-67 negatively stained nuclei (purple color). (**C**) Representative histological sections (40×) and mean values and standard deviation of TUNEL in the subcutaneous tumors of the non-treated control mice and the treated lung cancer animals. Black arrowheads point towards TUNEL-positively stained nuclei (brown color), while red arrowheads point towards TUNEL-negatively stained nuclei (purple color). Statistical significance: *: *p* ≤ 0.05 and ***: *p* ≤ 0.001 between lung cancer mice compared to the lung cancer-monoclonal antibodies group.

As illustrated in Table 2 and Figure 2A, by the end day (30) of the study protocol, in the lung cancer mice compared to non-treated control mice, treatment with the cocktail of monoclonal antibodies had significantly improved the following variables: final body weight, body weight gain with and without tumor, tumor weight (34% reduction), and tumor area (64% reduction). Importantly, levels of Ki-67-positive nuclei were significantly lower (27% reduction), while TUNEL-positively stained nuclei were significantly higher (127% increase) in the tumors of mice treated with the monoclonal antibodies compared to those detected in the non-treated control animals (Figure 2B,C).

### 3.2. Immune Microenvironment in Response to the Immunomodulators

**Figure 3:**
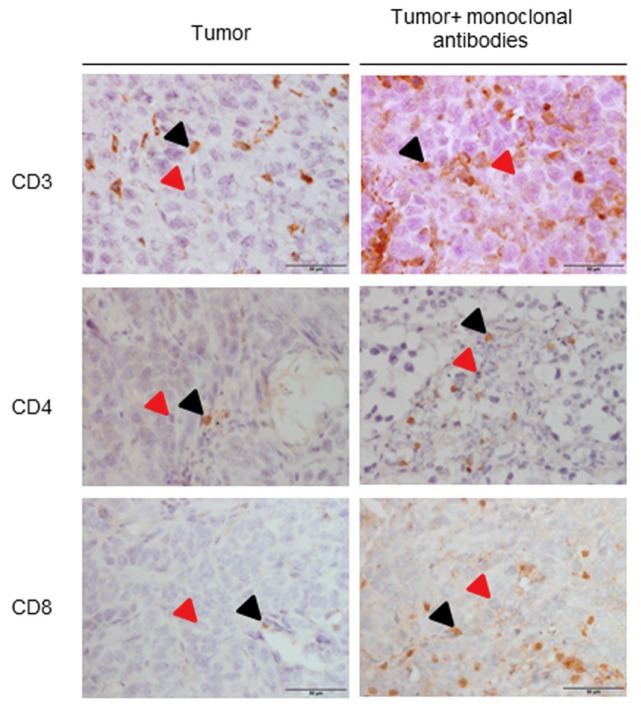
Representative examples of immunohistochemistry staining for CD3, CD4, and CD8, in tumor samples of the different study groups of mice. All types of T cells (CD3, CD8, and CD4) are stained in brown color (black arrows), while negative nuclei are stained in purple color (red arrows).

In tumors of mice treated with the monoclonal antibodies, the number of T cells (CD3, CD8, and CD4) was significantly greater than that observed in the non-treated animals (Table 3 and Figure 3).

### 3.3. Tumor Oxidative Stress in Response to the Immunomodulators

**Figure 4:**
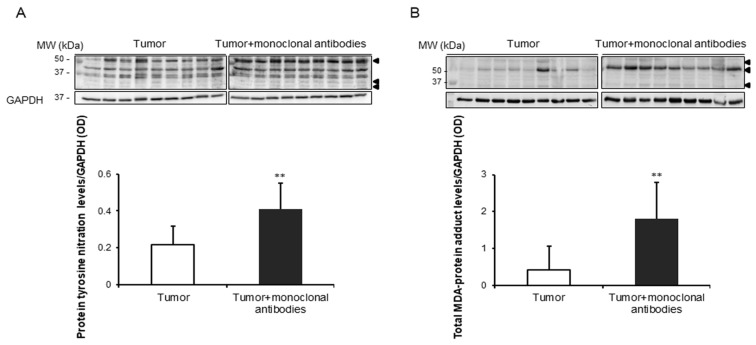
(**A**) Representative immunoblots and mean values and standard deviation of total protein tyrosine nitration levels/GAPDH in subcutaneous tumors of lung cancer mice as measured by optical densities. (**B**) Representative immunoblots and mean values and standard deviation of total MDA protein adduct levels/GAPDH in subcutaneous tumors of lung cancer mice as measured by optical densities. Representative GAPDH is shown as the loading control. Statistical significance is represented as follows: **: *p* ≤ 0.01 between non-treated controls (*N* = 9) in white bars and treated lung cancer (*N* = 9) mice in black bars. Definition of abbreviations: MDA, malondialdehyde; GAPDH, glyceraldehyde-3-phospate dehydrogenase; OD, optical densities.

**Figure 5:**
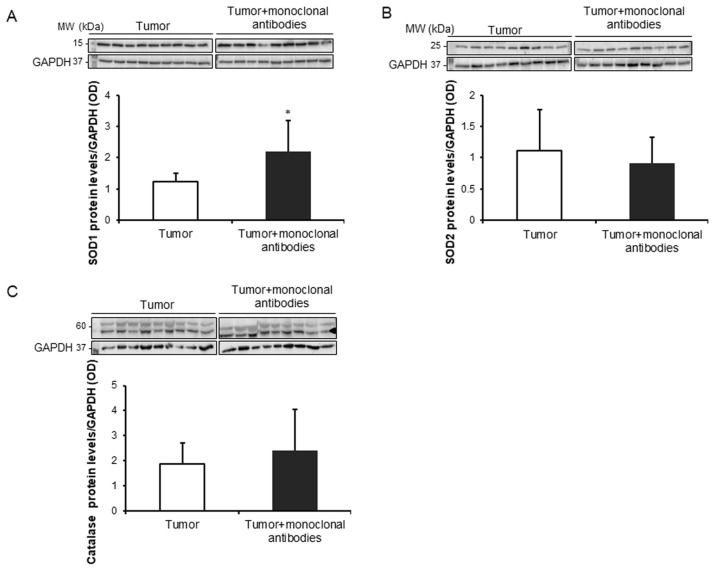
(**A**) Representative immunoblots and mean values and standard deviation of SOD1 protein levels/GAPDH in subcutaneous tumors of lung cancer mice as measured by optical densities. (**B**) Representative immunoblots and mean values and standard deviation of SOD2 protein levels/GAPDH in subcutaneous tumors of lung cancer (LC) mice as measured by optical densities. (**C**) Mean values and standard deviation of catalase protein levels/GAPDH in subcutaneous tumors of lung cancer mice as measured by optical densities. Representative GAPDH is shown as the loading control. Statistical significance is represented as follows: *: *p* ≤ 0.05 between non-treated controls (*N* = 9) in white bars and treated lung cancer (*N* = 9) mice in black bars. Definition of abbreviations: SOD1, CuZn-superoxide dismutase; SOD2, Mn-superoxide dismutase; GAPDH, glyceraldehyde-3-phospate dehydrogenase; OD, optical densities.

Compared to non-treated mice, protein tyrosine nitration and oxidation (MDA-protein adducts) and cytosolic SOD1 levels were significantly greater in the tumors of the mice treated with the monoclonal antibodies, while no significant differences were detected in mitochondrial SOD2 or catalase protein levels between the two study groups (Figure 4A,B and Figure 5A–C).

### 3.4. Tumor Apoptosis and Autophagy Markers in Response to Immunomodulators

**Figure 6:**
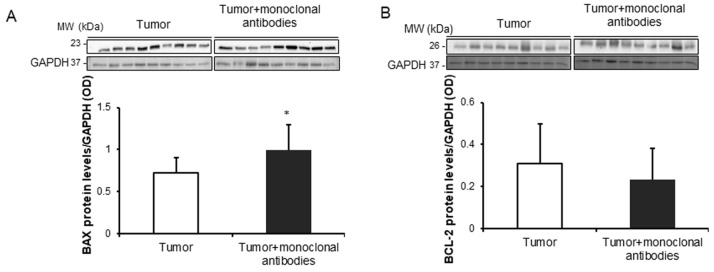
(**A**) Representative immunoblots and mean values and standard deviation of BAX protein levels/GAPDH in subcutaneous tumors of lung cancer mice as measured by optical densities. (**B**) Representative immunoblots and mean values and standard deviation of BCL-2 protein levels/GAPDH in subcutaneous tumors of LC mice as measured by optical densities. Representative GAPDH is shown as the loading control. Statistical significance is represented as follows: *: *p* ≤ 0.05 between non-treated controls (*N* = 9) in white bars and treated lung cancer (*N* = 9) mice in black bars. Definition of abbreviations: BAX, BCL-2 associated X protein; BCL-2, b-cell lymphoma 2; GAPDH, glyceraldehyde-3-phospate dehydrogenase; OD, optical densities.

**Figure 7:**
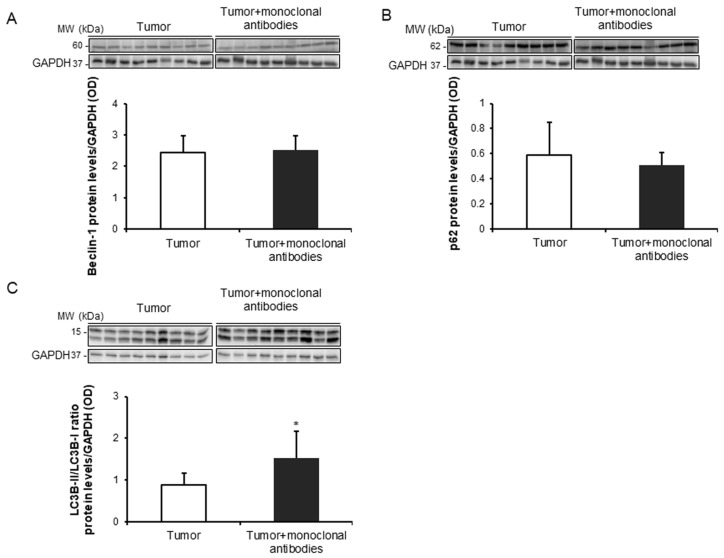
(**A**) Representative immunoblots and mean values and standard deviation of Beclin-1 protein levels/GAPDH in subcutaneous tumors of LC mice as measured by optical densities. (**B**) Representative immunoblots and mean values and standard deviation of p62 protein levels/GAPDH in subcutaneous tumors of LC mice as measured by optical densities. (**C**) Representative immunoblots and mean values and standard deviation of total LC3-II/LC3-I protein/GAPDH in subcutaneous tumors of LC mice as measured by optical densities. Representative GAPDH is shown as the loading control. Statistical significance is represented as follows: *: *p* ≤ 0.05 between non-treated controls (*N* = 9) in white bars and treated lung cancer (*N* = 9) mice in black bars. Definition of abbreviations: p62, nucleoporin p62; LC3, microtube-associated protein 1 light chain 3; GAPDH, glyceraldehyde-3-phospate dehydrogenase; OD, optical densities.

Treatment of the mice with the cocktail of monoclonal antibodies induced a significant rise in BAX protein levels in the tumors compared to non-treated animals, while no significant differences were found in tumor BCL-2 protein levels between the two study groups (Figure 6A,B). Treatment with monoclonal antibodies did not induce any significant difference in protein expression levels of either beclin-1 or p62, whereas the ratio of LC3-II to LC3-I was significantly increased in the tumors of the treated mice (Figure 7A–C).

### 3.5. Effects of the Immunomodulators on Signaling Markers in Tumors

**Figure 8:**
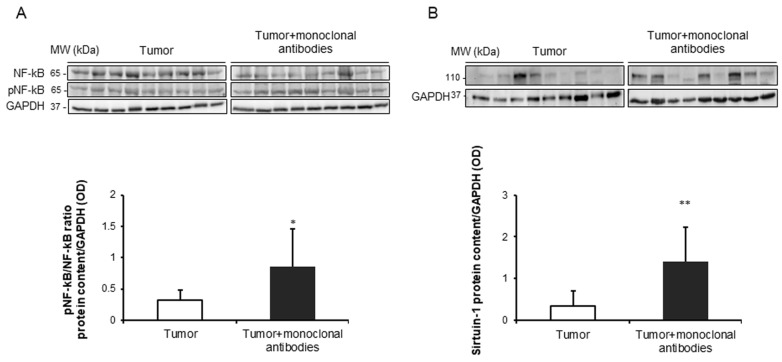
(**A**) Representative immunoblots and mean values and standard deviation of ratio of pNF-κB/NF-κB protein levels/GAPDH in subcutaneous tumors of lung cancer mice as measured by optical densities. (**B**) Representative immunoblots and mean values and standard deviation of sirtuin-1 protein levels/GAPDH in subcutaneous tumors of lung cancer mice as measured by optical densities. Representative GAPDH is shown as the loading control. Statistical significance is represented as follows: and *: *p* ≤ 0.05 and **: *p* ≤ 0.01 between non-treated controls (*N* = 9) in white bars and treated lung cancer (*N* = 9) mice in black bars. Definition of abbreviations: NF-κB, nuclear factor kappa-light-chain-enhancer of activated B cells; GAPDH, glyceraldehyde-3-phospate dehydrogenase; OD, optical densities.

Protein levels of the ratio of p-p65 subunit to total p65 of the NF-κB signaling pathway were significantly greater in the tumors of the mice treated with the monoclonal antibodies than in those of non-treated rodents (Figure 8A). Protein levels of the deacetylase sirtuin-1 were significantly increased in the tumors of the treated mice compared to non-treated animals (Figure 8B).

## 4. Discussion

In the present study, treatment of the tumor-bearing mice with a cocktail of monoclonal antibodies that specifically targeted immune checkpoints and pathways elicited a significant reduction of tumor proliferation rates and sizes through the induction of several biological mechanisms that are discussed below. Thus, the study hypothesis has been confirmed to a great extent.

In the tumors of the mice treated with the cocktail of monoclonal antibodies, the area was significantly reduced at the end of the study period. These are relevant findings that confirm the efficacy of the treatment with the immunomodulators of the tumor cells in this experimental model of lung cancer in mice. These results are in line with those previously reported, in which a complete regression of the tumors (melanoma and lung cancer) was attained following intratumoral treatment of the mice with the same cocktail of monoclonal antibodies [18,19]. In the present study, the numbers of T cell subtypes also significantly increased in the tumors of the mice that received treatment with the checkpoint inhibitors.

As opposed to previous investigations [18,19], the immunomodulators were injected intraperitoneally with the aim to mimic the treatments applied in patients, in whom drugs are usually administered systemically. In this investigation, a complete regression of the tumors was not achieved, probably as a result of the systemic administration of the drugs compared to previous reports [18,19], in which the monoclonal antibodies were administered locally. Nonetheless, in the tumor-bearing mice that received the medical treatment with the immunomodulators a substantial reduction in tumor burden as measured by both tumor weight (34%) and area (64%) was observed. This leads to the conclusion that the treatment, the doses, and the route were effectively administered in the current study.

Interestingly, the number of tumor proliferating cells was also significantly lower in the tumors of the mice treated with the monoclonal antibodies than in the non-treated tumor-bearing rodents. These results suggest that cell cycle arrest probably due to alterations in cyclin expression levels may account for the reduced levels of Ki-67-positively stained nuclei encountered in the adenocarcinoma cells of the treated tumor-bearing mice. Moreover, these findings are also in agreement with previous investigations, in which expression levels of Ki-67 were significantly reduced (34%) in the tumors of mice treated with several selective inhibitors of cell survival pathways [12], in those from transgenic mice deficient for either poly(Adenosine Diphosphate Ribose (ADP)-ribose)) polymerases (PARP)-1 or -2 enzymes [33], and in those of rodents treated with pharmacological inhibitors of PARP activity [44]. Taken together, these results are also very consistent with the findings reported herein: reduced tumor area and weight in the tumor-bearing mice treated with the immunomodulators at the end of the study period.

Oxidative stress was assessed using several indirect markers in the tumor cells of both groups of mice. Importantly, levels of protein tyrosine nitration and total MDA-protein adducts were significantly greater in the tumor cells of the mice that received treatment with the monoclonal antibodies. These results are in agreement with those previously observed in another investigation, in which protein oxidation levels were also increased in the tumors of Parp-1^-/-^ and Parp-2^-/-^ mice [33]. In the present investigation, levels of the antioxidant enzyme SOD1, but not those of SOD2 or catalase, were significantly greater in the tumors of the lung cancer-bearing mice treated with the monoclonal antibodies. These results are in line with those encountered in the tumors of mice treated with the proteasome inhibitor bortezomib [12]. Furthermore, accumulation of reactive oxygen species (ROS) and glutathione depletion were also shown in tumor cells of mice with colorectal cancer [34], and an oxidative stress-associated mechanism of T cell activation was observed in the stroma of ovarian and colon tumor samples in patients as well [35,45,46].

The rise in the expression of cytosolic SOD1 levels may have been a response to counterbalance the deleterious effects of increased oxidative stress in the tumor cells as previously suggested [12]. Altogether, a rise in several oxidative stress markers was observed in the tumors of the mice treated with the cocktail of monoclonal antibodies. These findings may imply that in response to treatment with the immunomodulators, oxidative stress may drive cell cycle arrest and tumor cell death independently of the immune response [47].

Oxidative stress may also trigger several important cellular pathways such as cell death, apoptosis, and autophagy through signaling pathways such as the redox sensitive NF-κB pathway. In this regard, the ratio levels of active p65 (phosphorylated) to total p65 were greater in the tumors of animals treated with the monoclonal antibodies than in the non-treated mice. Importantly, levels of TUNEL-positive nuclei were also significantly increased in tumors of the mice treated with the immunomodulators. Additionally, Bax protein levels also increased in the tumors of mice treated with the monoclonal antibodies, while no differences in Bcl2 levels were seen between the study groups. These results are consistent with those previously reported, in which different therapeutic strategies also elicited a rise in proapoptotic markers [12,33]. The increase in apoptotic markers of cancer cells was also demonstrated in previous investigations in which the animals were treated with selective inhibitors of PARP activity [44,48,49,50].

A rise in the autophagy marker LC3B was observed in the tumor cells of the mice treated with the immunomodulators compared to non-treated control rodents. These results imply that autophagy may also mediate the reduced tumor burden observed in the mice that received treatment with the cocktail of monoclonal antibodies. In fact, similar results were previously demonstrated in the tumors of mice that were genetically deficient for either PARP-1 or PARP-2 proteins, especially the latter [33].

The deacetylase sirtuin-1 may play a role in autophagy as a result of its upstream regulation of LC3B [51]. In the current study, a significant rise in protein levels of sirtuin-1 was detected in the tumor cells of the mice treated with the monoclonal antibodies compared to non-treated animals. On the other hand, sirtuin-1 may also play a role in the regulation of tumor microenvironment of the immune cells [52]. These results imply that sirtuin-1 probably interfered with immune cells [52], leading to changes in the tumor microenvironment (from Th2 type to Th1 immunity) as previously demonstrated [18,19]. This may further contribute to the reduced tumor burden observed in the mice treated with the monoclonal antibodies. Despite the relevance of this question, it will have to be fully elucidated in future investigations as it was clearly beyond the objectives of the current investigation.

### Study Limitations

Limitations inherent to the use of an animal experimental model may have occurred in the current investigation as compared to clinical studies. This may partly preclude the generalization of the present study results to clinical settings. Moreover, other limitations may be related to the type of tumor cells and the animal background as well as the type of laboratory techniques employed to identify the different immune cells compared to previous investigations [18,19]. Procedures beyond the histology, such as flow cytometry, may be useful to selectively identify the type and number of the cells contained in the tumors. Nonetheless, these experiments would have required a completely different experimental approach at the time of conducting the animal experiments and when collecting the tumor specimens. On the other hand, the histological approach enabled us to identify topographically the presence of the T cells within the cancer specimens, thus confirming that they were, indeed, part of the tumor microenvironment.

Another possible limitation in the study would be related to the lack of additional control groups of mice, such as animals administered with isotype-matched antibodies to confirm the selectivity and specificity of the immunotherapy. Nevertheless, as the efficacy and selectivity of the antibodies used in the present investigation had already been demonstrated in previous studies [18,19] and for ethical reasons, no additional control groups were included. Despite all these limitations, the experiments reported herein shed light onto novel mechanisms whereby immunotherapy may exert beneficial effects in lung adenocarcinoma tumors.

### 5. Conclusions

We have demonstrated that immunomodulators with different mechanisms of action elicited a reduction in the tumor burden as measured by tumor size and weight through several biological mechanisms, namely decreased tumor proliferation rates and increased T cell counts, oxidative stress, apoptosis, autophagy, and signaling pathways, which may have interfered with the immune profile of the tumor microenvironment. Future research should be devoted to the elucidation of the specific contribution of each mechanism (reduced tumor proliferation, increased tumor degradation, and stimulation of the immune tumor microenvironment) to the reduced tumor burden seen in this animal model of lung cancer. These findings may have potential therapeutic implications in patients under treatment with immunomodulators for their lung neoplasms.

## Figures and Tables

**Table 1:** Monoclonal antibodies for the treatment of lung tumors in mice.

Monoclonal Antibodies	Targets	Function
Anti-PD-1	PD-1	PD-1 receptor is expressed in activated T cells and induces immune tolerance by repressing T cell effector function [23,24].
Anti-CTLA-4	CTLA-4	CTLA-4 receptor is expressed in T cells and induces immune tolerance by repressing antigen presentation [23,25].
Anti-CD19	CD-19	CD-19 activates B cells [23,26].
Anti-CD137	CD-137	CD-137 receptor activates CD8^+^ T and NK cells [21,23].

**Table 2:** Physiological and tumor characteristics in the study groups of mice.

Variables	Lung Cancer Mice	Lung Cancer + Monoclonal Antibodies Mice
Initial body weight (g)	20.41 (1.22)	20.34 (0.79)
Final body weight (g)	19.35 (2.25)	21.39 (1.57), *
Body weight gain (%)	−4.27 (10.47)	+5.16 (6.33), *
Body weight gain without tumor (%)	−15.06 (11.28)	−2.66 (8.35), *
Tumor weight (g)	2.38 (0.75)	1.57 (0.89), *

Variables are presented as mean (standard deviation). Statistical significance: * *p* ≤ 0.05 between the two study groups of mice.

**Table 3:** Immune microenvironment in the study groups of mice.

T Cells	Lung Cancer Mice	Lung Cancer + Monoclonal Antibodies Mice
CD3 + cells (%)	8.34 (0.91)	11.30 (0.78), ***
CD4 + (cells/µm^2^)	1.99 × 10^−6^ (0.44 × 10^−6^)	3.37 × 10^−6^ (1.49 × 10^−6^), *
CD8 + cells (%)	5.16 (1.35)	7.86 (1.04), ***

Values are expressed as mean (standard deviation). Statistical significance: * *p* ≤ 0.05 and *** *p* ≤ 0.001 between the two experimental groups of mice.

## References

[1] Siegel R.L., Miller K.D., Jemal A. (2018). Cancer statistics, 2018. CA Cancer J. Clin..

[2] Alvarez F.V., Trueba I.M., Sanchis J.B., Lopez-Rodo L.M., Rodriguez Suarez P.M., de Cos Escuin J.S., Barreiro E., Henar Borrego P.M., Vicente C.D., Aldeyturriaga J.F. (2016). Recommendations of the Spanish Society of Pneumology and Thoracic Surgery on the diagnosis and treatment of non-small-cell lung cancer. Arch. Bronconeumol..

[3] Malvezzi M., Carioli G., Bertuccio P., Boffetta P., Levi F., La V.C., Negri E. (2017). European cancer mortality predictions for the year 2017, with focus on lung cancer. Ann. Oncol..

[4] Miravitlles M., Soler-Cataluna J.J., Calle M., Molina J., Almagro P., Quintano J.A., Trigueros J.A., Cosio B.G., Casanova C., Antonio R.J. (2017). Spanish Guidelines for Management of Chronic Obstructive Pulmonary Disease (GesEPOC) 2017. Pharmacological Treatment of Stable Phase. Arch. Bronconeumol..

[5] Vogelmeier C.F., Criner G.J., Martinez F.J., Anzueto A., Barnes P.J., Bourbeau J., Celli B.R., Chen R., Decramer M., Fabbri L.M. (2017). Global Strategy for the Diagnosis, Management, and Prevention of Chronic Obstructive Lung Disease 2017 Report: GOLD Executive Summary. Arch. Bronconeumol..

[6] Álvarez F.V., Trueba I.M., Sanchis J.B., López-Rodó L.M., Suárez P.M.R., de Cos Escuin J.S., Barreiro E., Pintado M.H.B., Vicente C.D., Aldeyturriaga J.F. (2016). Executive summary of the SEPAR recommendations for the diagnosis and treatment of non-small cell lung cancer. Arch. Bronconeumol..

[7] Conway E.M., Pikor L.A., Kung S.H., Hamilton M.J., Lam S., Lam W.L., Bennewith K.L. (2016). Macrophages, Inflammation, and Lung Cancer. Am. J. Respir. Crit. Care Med..

[8] Mateu-Jimenez M., Curull V., Pijuan L., Sanchez-Font A., Rivera-Ramos H., Rodriguez-Fuster A., Aguilo R., Gea J., Barreiro E. (2017). Systemic and Tumor Th1 and Th2 Inflammatory Profile and Macrophages in Lung Cancer: Influence of Underlying Chronic Respiratory Disease. J. Thorac. Oncol..

[9] O’Byrne K.J., Dalgleish A.G. (2001). Chronic immune activation and inflammation as the cause of malignancy. Br. J. Cancer.

[10] O’Callaghan D.S., O’Donnell D., O’Connell F., O’Byrne K.J. (2010). The role of inflammation in the pathogenesis of non-small cell lung cancer. J. Thorac. Oncol..

[11] Barreiro E., Fermoselle C., Mateu-Jimenez M., Sanchez-Font A., Pijuan L., Gea J., Curull V. (2013). Oxidative stress and inflammation in the normal airways and blood of patients with lung cancer and COPD. Free Radic. Biol. Med..

[12] Mateu-Jimenez M., Fermoselle C., Rojo F., Mateu J., Pena R., Urtreger A.J., Diament M.J., Joffe E.D., Pijuan L., Herreros A.G. (2016). Pharmacological Approaches in an Experimental Model of Non-Small Cell Lung Cancer: Effects on Tumor Biology. Curr. Pharm. Des..

[13] Mateu-Jimenez M., Sanchez-Font A., Rodriguez-Fuster A., Aguilo R., Pijuan L., Fermoselle C., Gea J., Curull V., Barreiro E. (2016). Redox Imbalance in Lung Cancer of Patients with Underlying Chronic Respiratory Conditions. Mol. Med..

[14] Barreiro E., Bustamante V., Curull V., Gea J., Lopez-Campos J.L., Munoz X. (2016). Relationships between chronic obstructive pulmonary disease and lung cancer: Biological insights. J. Thorac. Dis..

[15] Pore M.M., Hiltermann T.J., Kruyt F.A. (2013). Targeting apoptosis pathways in lung cancer. Cancer Lett..

[16] Mateu-Jimenez M., Curull V., Rodriguez-Fuster A., Aguilo R., Sanchez-Font A., Pijuan L., Gea J., Barreiro E. (2018). Profile of epigenetic mechanisms in lung tumors of patients with underlying chronic respiratory conditions. Clin. Epigenet..

[17] Schreiber R.D., Old L.J., Smyth M.J. (2011). Cancer immunoediting: Integrating immunity’s roles in cancer suppression and promotion. Science.

[18] Dai M., Wei H., Yip Y.Y., Feng Q., He K., Popov V., Hellstrom I., Hellstrom K.E. (2013). Long-lasting complete regression of established mouse tumors by counteracting Th2 inflammation. J. Immunother..

[19] Dai M., Yip Y.Y., Hellstrom I., Hellstrom K.E. (2015). Curing mice with large tumors by locally delivering combinations of immunomodulatory antibodies. Clin. Cancer Res..

[20] Tsou P., Katayama H., Ostrin E.J., Hanash S.M. (2016). The Emerging Role of B Cells in Tumor Immunity. Cancer Res..

[21] Yonezawa A., Dutt S., Chester C., Kim J., Kohrt H.E. (2015). Boosting Cancer Immunotherapy with Anti-CD137 Antibody Therapy. Clin. Cancer Res..

[22] Chen D.S., Mellman I. (2017). Elements of cancer immunity and the cancer-immune set point. Nature.

[23] Chen D.S., Mellman I. (2013). Oncology meets immunology: The cancer-immunity cycle. Immunity.

[24] Jin H.T., Ahmed R., Okazaki T. (2011). Role of PD-1 in regulating T-cell immunity. Curr. Top. Microbiol. Immunol..

[25] Selby M.J., Engelhardt J.J., Quigley M., Henning K.A., Chen T., Srinivasan M., Korman A.J. (2013). Anti-CTLA-4 antibodies of IgG2a isotype enhance antitumor activity through reduction of intratumoral regulatory T cells. Cancer Immunol. Res..

[26] Forsthuber T.G., Cimbora D.M., Ratchford J.N., Katz E., Stuve O. (2018). B cell-based therapies in CNS autoimmunity: Differentiating CD19 and CD20 as therapeutic targets. Ther. Adv. Neurol. Disord..

[27] Blank C.U., Enk A. (2015). Therapeutic use of anti-CTLA-4 antibodies. Int. Immunol..

[28] Haanen J.B., Robert C. (2015). Immune Checkpoint Inhibitors. Prog. Tumor Res..

[29] Rolfo C., Caglevic C., Santarpia M., Araujo A., Giovannetti E., Gallardo C.D., Pauwels P., Mahave M. (2017). Immunotherapy in NSCLC: A Promising and Revolutionary Weapon. Adv. Exp. Med. Biol..

[30] Fernandez-Bussy S., Pires Y., Labarca G., Vial M.R. (2018). PD-L1 Expression in a Non-Small Cell Lung Cancer Specimen Obtained by EBUS-TBNA. Arch. Bronconeumol..

[31] Moliner L., Fernandez C., Clave S., Arriola E. (2019). Accurate Identification of Predictive Biomarkers of Response to Targeted Therapies in Lung Cancer with Next Generation Sequencing. Arch. Bronconeumol..

[32] Rocha P., Arriola E. (2019). Immunotherapy is Here to Stay: A New Treatment Paradigm in Lung Cancer. Arch. Bronconeumol..

[33] Mateu-Jimenez M., Cucarull-Martinez B., Yelamos J., Barreiro E. (2016). Reduced tumor burden through increased oxidative stress in lung adenocarcinoma cells of PARP-1 and PARP-2 knockout mice. Biochimie.

[34] Habtetsion T., Ding Z.C., Pi W., Li T., Lu C., Chen T., Xi C., Spartz H., Liu K., Hao Z. (2018). Alteration of Tumor Metabolism by CD4+ T Cells Leads to TNF-alpha-Dependent Intensification of Oxidative Stress and Tumor Cell Death. Cell Metab..

[35] Maj T., Wang W., Crespo J., Zhang H., Wang W., Wei S., Zhao L., Vatan L., Shao I., Szeliga W. (2017). Oxidative stress controls regulatory T cell apoptosis and suppressor activity and PD-L1-blockade resistance in tumor. Nat. Immunol..

[36] Chacon-Cabrera A., Fermoselle C., Urtreger A.J., Mateu-Jimenez M., Diament M.J., de Kier Joffe E.D., Sandri M., Barreiro E. (2014). Pharmacological strategies in lung cancer-induced cachexia: Effects on muscle proteolysis, autophagy, structure, and weakness. J. Cell Physiol..

[37] Chacon-Cabrera A., Fermoselle C., Salmela I., Yelamos J., Barreiro E. (2015). MicroRNA expression and protein acetylation pattern in respiratory and limb muscles of Parp-1(−/−) and Parp-2(−/−) mice with lung cancer cachexia. Biochim. Biophys. Acta.

[38] Chacon-Cabrera A., Mateu-Jimenez M., Langohr K., Fermoselle C., Garcia-Arumi E., Andreu A.L., Yelamos J., Barreiro E. (2017). Role of Parp Activity in Lung Cancer-induced Cachexia: Effects on Muscle Oxidative Stress, Proteolysis, Anabolic Markers and Phenotype. J. Cell Physiol..

[39] Diament M.J., Garcia C., Stillitani I., Saavedra V.M., Manzur T., Vauthay L., Klein S. (1998). Spontaneous murine lung adenocarcinoma (P07): A new experimental model to study paraneoplastic syndromes of lung cancer. Int. J. Mol. Med..

[40] Diament M.J., Peluffo G.D., Stillitani I., Cerchietti L.C., Navigante A., Ranuncolo S.M., Klein S.M. (2006). Inhibition of tumor progression and paraneoplastic syndrome development in a murine lung adenocarcinoma by medroxyprogesterone acetate and indomethacin. Cancer Invest..

[41] Urtreger A.J., Diament M.J., Ranuncolo S.M., Vidal D.C., Puricelli L.I., Klein S.M., Bal de Kier Joffe E.D. (2001). New murine cell line derived from a spontaneous lung tumor induces paraneoplastic syndromes. Int. J. Oncol..

[42] Chacon-Cabrera A., Gea J., Barreiro E. (2016). Short- and Long-Term Hindlimb Immobilization and Reloading: Profile of Epigenetic Events in Gastrocnemius. J. Cell Physiol..

[43] Salazar-Degracia A., Granado-Martinez P., Millan-Sanchez A., Tang J., Pons-Carreto A., Barreiro E. (2019). Reduced lung cancer burden by selective immunomodulators elicits improvements in muscle proteolysis and strength in cachectic mice. J. Cell Physiol..

[44] Albert J.M., Cao C., Kim K.W., Willey C.D., Geng L., Xiao D., Wang H., Sandler A., Johnson D.H., Colevas A.D. (2007). Inhibition of poly(ADP-ribose) polymerase enhances cell death and improves tumor growth delay in irradiated lung cancer models. Clin. Cancer Res..

[45] Kryczek I., Wang L., Wu K., Li W., Zhao E., Cui T., Wei S., Liu Y., Wang Y., Vatan L. (2016). Inflammatory regulatory T cells in the microenvironments of ulcerative colitis and colon carcinoma. Oncoimmunology.

[46] Wang W., Kryczek I., Dostal L., Lin H., Tan L., Zhao L., Lu F., Wei S., Maj T., Peng D. (2016). Effector T Cells Abrogate Stroma-Mediated Chemoresistance in Ovarian Cancer. Cell.

[47] Su X., Jiang X., Meng L., Dong X., Shen Y., Xin Y. (2018). Anticancer Activity of Sulforaphane. The Epigenetic Mechanisms and the Nrf2 Signaling Pathway. Oxid. Med. Cell Longev..

[48] Gangopadhyay N.N., Luketich J.D., Opest A., Visus C., Meyer E.M., Landreneau R., Schuchert M.J. (2011). Inhibition of poly(ADP-ribose) polymerase (PARP) induces apoptosis in lung cancer cell lines. Cancer Invest..

[49] Gaymes T.J., Shall S., MacPherson L.J., Twine N.A., Lea N.C., Farzaneh F., Mufti G.J. (2009). Inhibitors of poly ADP-ribose polymerase (PARP) induce apoptosis of myeloid leukemic cells: Potential for therapy of myeloid leukemia and myelodysplastic syndromes. Haematologica.

[50] Karpel-Massler G., Pareja F., Aime P., Shu C., Chau L., Westhoff M.A., Halatsch M.E., Crary J.F., Canoll P., Siegelin M.D. (2014). PARP inhibition restores extrinsic apoptotic sensitivity in glioblastoma. PLoS ONE.

[51] Huang R., Xu Y., Wan W., Shou X., Qian J., You Z., Liu B., Chang C., Zhou T., Lippincott-Schwartz J. (2015). Deacetylation of nuclear LC3 drives autophagy initiation under starvation. Mol. Cell.

[52] Arts R.J., Huang P.K., Yang D., Joosten L.A., Van Der Meer J.W., Oppenheim J.J., Netea M.G., Cheng S.C. (2018). High-Mobility Group Nucleosome-Binding Protein 1 as Endogenous Ligand Induces Innate Immune Tolerance in a TLR4-Sirtuin-1 Dependent Manner in Human Blood Peripheral Mononuclear Cells. Front. Immunol..

